# Rapid Determination of Myosin Heavy Chain Expression in Rat, Mouse, and Human Skeletal Muscle Using Multicolor Immunofluorescence Analysis

**DOI:** 10.1371/journal.pone.0035273

**Published:** 2012-04-18

**Authors:** Darin Bloemberg, Joe Quadrilatero

**Affiliations:** Department of Kinesiology, University of Waterloo, Waterloo, Ontario, Canada; University of Texas Health Science Center at Houston, United States of America

## Abstract

Skeletal muscle is a heterogeneous tissue comprised of fibers with different morphological, functional, and metabolic properties. Different muscles contain varying proportions of fiber types; therefore, accurate identification is important. A number of histochemical methods are used to determine muscle fiber type; however, these techniques have several disadvantages. Immunofluorescence analysis is a sensitive method that allows for simultaneous evaluation of multiple MHC isoforms on a large number of fibers on a single cross-section, and offers a more precise means of identifying fiber types. In this investigation we characterized pure and hybrid fiber type distribution in 10 rat and 10 mouse skeletal muscles, as well as human vastus lateralis (VL) using multicolor immunofluorescence analysis. In addition, we determined fiber type-specific cross-sectional area (CSA), succinate dehydrogenase (SDH) activity, and α-glycerophosphate dehydrogenase (GPD) activity. Using this procedure we were able to easily identify pure and hybrid fiber populations in rat, mouse, and human muscle. Hybrid fibers were identified in all species and made up a significant portion of the total population in some rat and mouse muscles. For example, rat mixed gastrocnemius (MG) contained 12.2% hybrid fibers whereas mouse white tibialis anterior (WTA) contained 12.1% hybrid fibers. Collectively, we outline a simple and time-efficient method for determining MHC expression in skeletal muscle of multiple species. In addition, we provide a useful resource of the pure and hybrid fiber type distribution, fiber CSA, and relative fiber type-specific SDH and GPD activity in a number of rat and mouse muscles.

## Introduction

Skeletal muscle is a heterogeneous tissue containing fibers with diverse morphological and functional characteristics [1], [2]. Muscle fibers can be classified into groups based on properties such as contractile speed, myosin heavy chain (MHC) expression, and metabolic capacity [2], [3]. In general, adult mammalian muscle can contain 4 major MHC isoforms; one slow isoform (MHCI) and three fast isoforms (MHCIIa, MHCIIx, MHCIIb). Notably, human skeletal muscle does not contain MHCIIb [2], [4], [5]. As such, fibers expressing MHCI are termed type I fibers, whereas fibers expressing MHCIIa, MHCIIx, and MHCIIb are termed type IIA, type IIX, and type IIB fibers, respectively. In addition, “hybrid" fibers containing two MHC isoforms (i.e., type I/IIA, IIAX, IIXB) can also be present in muscle [4], [6]. See review by Schiaffino [7] for a recent perspective regarding skeletal muscle fiber types.

Several approaches have been used to identify muscle fiber types, including: 1) determining myosin ATPase activity based on differential responses to various pH levels [8]–[10], 2) evaluating both myosin ATPase and oxidative enzyme activities [11]–[13], and 3) using immunohistochemical procedures with antibodies against specific MHC isoforms [14], [15]. While useful, evaluating myosin ATPase activity alone or with additional metabolic enzyme histochemistry procedures has a number of disadvantages and limitations. In particular, this requires multiple muscle cross-sections and incubations, and differences in the pattern of myosin ATPase inactivation following pH pre-incubations for the same fiber type have been noted across species [16]. Standard immunohistochemistry procedures using immunoperoxidase staining are also limited by the need for serial cross-sections to evaluate multiple MHC isoforms. Ultimately, these procedures require a significant time-investment, and do not easily allow for the identification of hybrid fibers. The purpose of this investigation was to: 1) outline a simple and time-efficient immunofluorescence staining protocol for determining MHC expression in rat, mouse, and human skeletal muscle, 2) evaluate the utility of a commercially available MHCIIx antibody in several species, 3) determine the pure (type I, IIA, IIX, IIB) and hybrid (type I/IIA, IIAX, IIXB) fiber composition in 1 human, 10 rat, and 10 mouse muscles, and 4) characterize fiber type-specific oxidative potential, glycolytic potential, and cross-sectional area (CSA) in rat, mouse, and human muscle.

## Materials and Methods

### Animals

Male Sprague-Dawley rats (n = 6; age: 21.7±0.5 weeks; weight: 458.2±4.8 g) purchased from Harlan (Indianapolis), and male C57BL/6 mice (n = 6; age: 23.0±0.9 weeks; weight: 28.5±1.2 g) from an in-house breeding colony were group housed on a 12∶12 hr reverse light/dark cycle in a temperature and humidity controlled environment. Standard lab chow and tap water were provided ad libitum. All animal procedures were approved by the University of Waterloo Animal Care Committee (AUPP 09-21).

### Muscle Preparation

Muscles were removed according to the scheme outlined by Armstrong and Phelps [11]. Muscles were chosen because of their common use in muscle biology and exercise physiology research. Whole soleus (Sol), plantaris (Pla), and extensor digitorum longus (EDL) were removed and a portion of the entire circumference around the mid-belly was used. The tibialis anterior was separated into red (RTA) and white (WTA) portions, the gastrocnemius was separated into red (RG), white (WG), and mixed (MG) portions, whereas the vastus intermedius (VI) and the white vastus lateralis (WVL) were isolated from the quadriceps. This resulted in a total of ten rat and mouse muscles/muscle portions. Muscles were embedded in O.C.T. compound (Tissue-Tek), frozen in liquid nitrogen-cooled isopentane, stored at −80°C, and cut into 10 µm thick cryosections with a cryostat (Thermo Electronic) maintained at −20°C. Human vastus lateralis (VL) muscle samples from recreationally active males (n = 7; age: 20.6±0.6 years; height: 183.4±0.6 cm; weight: 72.6±3.3 kg) from a recent report [17] were also utilized. Human procedures were approved by the University of Guelph Research Ethics Board (REB# 06MR027).

### Immunofluorescence and Histochemical Analysis

Immunofluorescence analysis of MHC expression was performed with primary antibodies against MHCI (BA-F8), MHCIIa (SC-71, 2F7), MHCIIx (6H1), MHCIIb (BF-F3), and all MHC isoforms except MHCIIx (BF-35) [14], [15]. Primary antibodies were purchased from the Developmental Studies Hybridoma Bank (University of Iowa), whereas secondary antibodies were purchased from Invitrogen. See Table 1 and Table 2 for antibody cocktail configurations and immunofluorescence staining procedures, respectively. Slides were visualized with an Axio Observer Z1 microscope (Carl Zeiss) using conventional widefield fluorescence microscopy as well as optical sectioning via structured-illumination fluorescence microscopy (Apotome, Carl Zeiss). The microscope was equipped with Red (Excitation: BP 545/25 nm; Emission BP 605/70 nm), Green (Excitation: BP 470/40 nm; Emission BP 525/50 nm), Blue (Excitation: BP 365/12 nm; Emission LP 397 nm) filters, an AxioCam HRm camera, and AxioVision software (Carl Zeiss). Individual images were taken across the entire cross-section and assembled into a composite panoramic image with Microsoft Image Composite Editor (Microsoft). For fiber type analysis, all fibers within the entire muscle/cross-section were characterized. Fiber CSA measurements for each fiber type were performed by outlining at least 40% of all fibers within a muscle/cross-section. Fiber counts, fiber type percentages, and CSA data are reported as group means ± SEM based on individual animal/subject means/values (rats: n = 6; mice: n = 6; humans: n = 7). Quantification was performed to assess relative fluorescence in previously categorized pure and hybrid fibers in a subset of muscles from each species. Pure and hybrid fiber fluorescence was determined (>20 fibers per type/sample) by subtracting the average fluorescence from unstained fibers (background) within a particular color channel. Fluorescence in pure fibers is expressed in arbitrary units (AU) and assigned a value of 1.0, with corresponding hybrid fibers expressed relative to the fluorescence obtained in the respective pure fibers.

**Table 1:** List of major antibodies and cocktail configurations used for MHC staining of rat, mouse, and human skeletal muscle.

Species	Primary Antibody Cocktails and Concentrations	MHC Reactivity	Secondary Antibody Cocktails and Concentrations
Rat and Mouse	BA-F8 (1∶50)	I	Alexa Fluor 350 IgG_2b_ 1∶500 (blue)
	SC-71 (1∶600)	IIa	Alexa Fluor 488 IgG_1_ 1∶500 (green)
	BF-F3 (1∶100)	IIb	Alexa Fluor 555 IgM 1∶500 (red)
Rat and Mouse	SC-71 (1∶600)	IIa	Alexa Fluor 488 IgG_1_ 1∶500 (green)
	6H1 (1∶50)	IIx	Alexa Fluor 555 IgM 1∶500 (red)^1^
Human	BA-F8 (1∶50)	I	Alexa Fluor 350 IgG_2b_ 1∶500 (blue)
	SC-71 (1∶600)	IIa+IIx*	Alexa Fluor 488 IgG_1_ 1∶500 (green)
	6H1 (1∶50)	IIx	Alexa Fluor 555 IgM 1∶500 (red)
Human	BA-F8 (1∶50)	I	Alexa Fluor 350 IgG_2b_ 1∶500 (blue)
	BF-35 (1∶100)	I+IIa	Alexa Fluor 488 IgG_1_ 1∶500 (green)
	6H1 (1∶50)	IIx	Alexa Fluor 555 IgM 1∶500 (red)

All antibody cocktails are prepared in block solution (10% goat serum in PBS).

* Non-specific cross-reactivity with MHCIIx was observed in human skeletal muscle.

1 Fibers positive for MHCIIx showed red staining, but for presentation and discussion purposes in rats and mice these fibers were pseudo-colored purple to avoid confusion with MHCIIb positive fibers.

**Table 2:** Overview of immunofluorescence MHC staining protocol.

Procedure	Time
Cut O.C.T.-embedded muscle into 10 µm cross-sections and store at −80°C	
Air dry sections (entire procedure performed at room temperature)	10 min
Block with 10% goat serum in PBS	60 min
Apply 1° antibody cocktail*	60 min (human, rat); 120 min (mouse)
PBS wash	3×5 min
Apply 2° antibody cocktail*	60 min
PBS wash	3×5 min
Mount coverslips with Prolong® Gold antifade reagent	

* See Table 1 for primary and secondary antibody cocktail configurations.

Histochemical staining for succinate dehydrogenase (SDH) [18], [19] and α-glycerophosphate dehydrogenase (GPD) [20] activity were determined as general indices of oxidative and glycolytic potential, respectively. For each enzyme, histochemical staining of all muscles or muscle portions for a given animal was performed simultaneously. Images were acquired with a brightfield Nikon microscope linked to a PixeLink digital camera and quantified with Image-Pro PLUS analysis software (Image-Pro PLUS). Individual images were assembled into composite panoramic images and matched to images obtained in the MHC analysis. SDH and GPD activity staining were determined on the same fibers analysed for CSA, and calculated by subtracting the background from 3–4 areas on each slide. Data are expressed relative to the values obtained in type I fibers (soleus for rat and mouse, VL for human), assigned a reference value of 1.0, and reported as mean optical density in AU. Fiber counts, SDH, and GPD data are reported as group means ± SEM based on individual animal/subject means/values (rats: n = 6; mice: n = 6; humans: n = 7).

## Results

### Rat and Mouse Muscle MHC Expression

**Figure 1:**
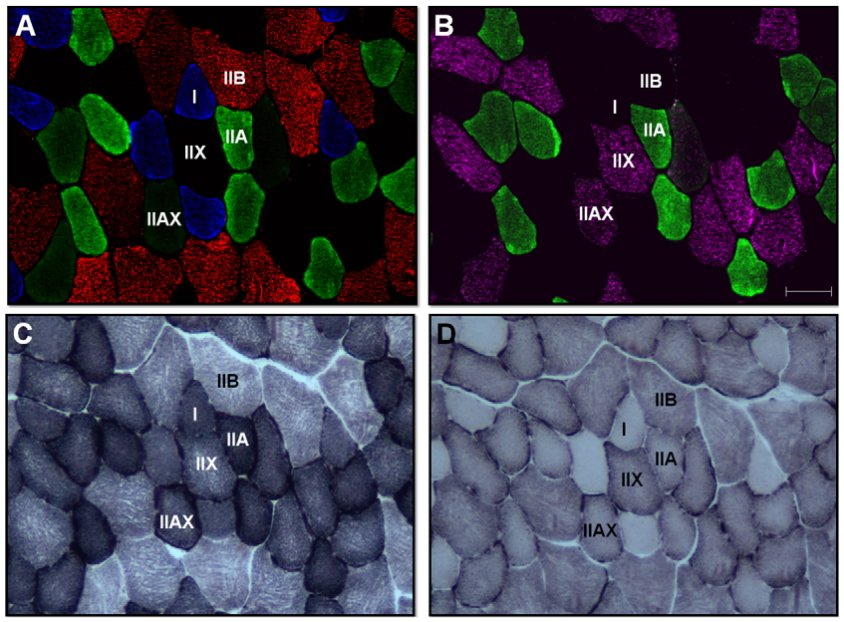
Representative images of rat red tibialis anterior (RTA) muscle showing MHC expression as well as SDH and GPD activity staining. Panel A, rat muscle serial cross-section incubated with a primary antibody cocktail against MHCI (BA-F8), MHCIIa (SC-71), and MHCIIb (BF-F3), followed by incubation with appropriate fluorescent-conjugated secondary antibodies. Shown are type I (blue), type IIA (green), type IIB (red), type IIX (unstained), and type IIAX (intermediate green) fibers. Panel B, rat muscle serial cross-section incubated with a primary antibody cocktail against MHCIIa (SC-71) and MHCIIx (6H1), followed by incubation with appropriate fluorescent-conjugated secondary antibodies. This confirms the presence of type IIA (green) fibers, as well as confirms that the unstained fibers and intermediate green stained fibers in Panel A are type IIX (purple) and type IIAX fibers (green and purple), respectively. Panel C, rat muscle serial cross-section showing SDH activity staining. Panel D, rat muscle serial cross-section showing GPD activity staining. Bar represents 50 µm.

**Figure 2:**
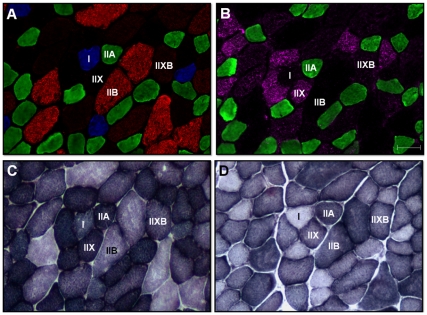
Representative images of mouse red gastrocnemius (RG) muscle showing MHC expression as well as SDH and GPD activity staining. Panel A, mouse muscle serial cross-section incubated with a primary antibody cocktail against MHCI (BA-F8), MHCIIa (SC-71), and MHCIIb (BF-F3), followed by incubation with appropriate fluorescent-conjugated secondary antibodies. Shown are type I (blue), type IIA (green), type IIB (red), type IIX (unstained), and type IIXB (intermediate red) fibers. Panel B, mouse muscle serial cross-section incubated with a primary antibody cocktail against MHCIIa (SC-71) and MHCIIx (6H1), followed by incubation with appropriate fluorescent-conjugated secondary antibodies. This confirms the presence of type IIA (green) fibers, as well as confirms that the unstained fibers and intermediate red stained fibers in Panel A are type IIX (purple) and type IIXB (purple and red) fibers, respectively. Panel C, mouse muscle serial cross-section showing SDH activity staining. Panel D, mouse muscle serial cross-section showing GPD activity staining. Bar represents 50 µm.

**Figure 3:**
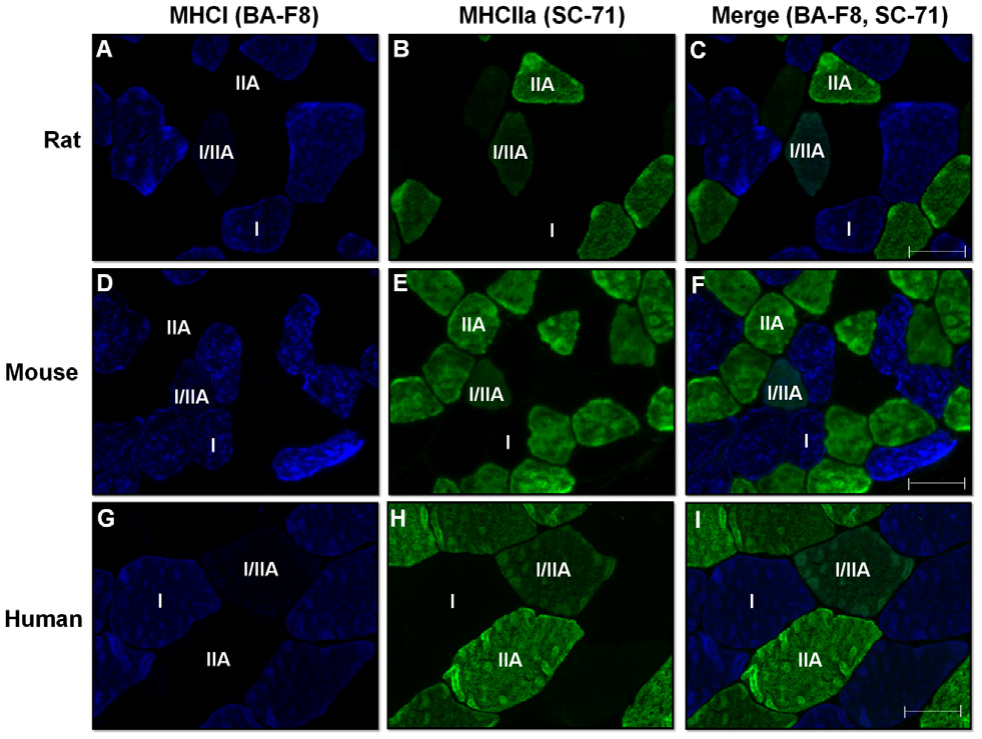
Representative images of rat, mouse, and human muscle showing type I/IIA hybrid fibers. Panels A–C (rat RG), D–F (mouse soleus), and G–I (human VL) are images of muscle cross-sections incubated concurrently with primary antibodies against MHCI (BA-F8) and MHCIIa (SC-71) showing type I (blue), type IIA (green), and type I/IIA (blue and green) fibers. Bars represent 50 µm.

Incubation of rat and mouse muscle cross-sections with an antibody cocktail resulted in positive staining of the same fibers as serial cross-sections incubated with individual antibodies (*Supplementary Figure S1*). In addition, strong fluorescent staining was observed in muscles incubated with primary and secondary antibody cocktails, whereas negligible staining was observed in control slides incubated with the secondary antibody cocktail only (*Supplementary Figure S2*). Therefore, all subsequent experiments were performed with antibody cocktails. Immunofluorescence analysis on a single muscle cross-section was able to easily identify the four major adult fiber types, notably type I (blue), type IIA (green), type IIB (red), and type IIX (unstained). In addition, we confirmed the identity of these unstained fibers as type IIX by staining serial cross-sections using an antibody specific for MHCIIx (*fibers positive for MHCIIx showed red staining, but for presentation and discussion purposes in rats and mice these fibers were pseudo-colored purple to avoid confusion with MHCIIb positive fibers*) (Figures 1 & 2). Our procedure also enabled identification of hybrid fibers containing two MHC isoforms (type I/IIA, IIAX, and IIXB) (Figures 1, 2, 3). Quantification of fluorescence intensity of previously categorized pure and hybrid fibers demonstrated that hybrid fibers had lower fluorescence intensity in both color channels relative to their respective pure fiber counterparts (*Supplementary Figure S3*). The level of fluorescence was above background values (as this was subtracted to give the net fluorescence), which demonstrates that our categorization of fibers was accurate.

Across 10 muscles we fiber typed an average of 11,553±407 fibers per rat (ranging from 583±50 fibers in WTA to 2827±211 fibers in plantaris) and 6,738±237 fibers per mouse (ranging from 542±78 fibers in WVL to 857±50 fibers in RTA). The fiber type composition for each rat and mouse muscle is given in Table 3 and Table 4, respectively. Of note is the relatively “slower" phenotype in corresponding muscles of rats compared to mice (i.e., soleus and RG). Hybrid fibers were found in all rat and mouse muscles. Fibers co-expressing MHCIIa and MHCIIx (type IIAX) were the most common hybrid fibers found in both rats and mice, followed by fibers co-expressing MHCIIx and MHCIIb (type IIXB) (Figures 1 & 2). Although fibers co-expressing MHCI and MHCIIa (type I/IIA) were relatively rare, they were found in all species (Figure 3).

**Table 3:** Quantitative analysis of fiber type distribution, CSA, SDH activity, and GPD activity in rat muscles.

			Fiber Type						
Rat Muscle		Fiber Count	Type I	Type I/IIA	Type IIA	Type IIAX	Type IIX	Type IIXB	Type IIB
**Red**	Population (%)	853±82	39.2±0.9	2.2±0.8	30.2±1.4	4.6±0.4	23.8±1.3	0	0
**Gastrocnemius**	CSA (µm^2^)	468±26	3337.6±55.0	3061.9±52.4	3260.6±55.8	3527.8±60.4	3754.7±64.3	-	-
**(RG)**	SDH (AU)	468±26	0.717±0.120	1.095±0.167	1.527±0.192	1.226±0.139	1.088±0.145	-	-
	GPD (AU)	468±26	0.595±0.078	1.160±0.309	2.004±0.143	2.771±0.302	3.402±0.313	-	-
**Mixed**	Population (%)	771±86	4.5±1.3	0	14.9±2.2	6.6±0.4	42.5±4.7	5.6±0.5	25.8±5.4
**Gastrocnemius**	CSA (µm^2^)	424±29	2069.8±39.5	-	2249.1±35.2	2835.4±45.0	3799.9±65.0	5595.6±88.8	6576.5±100.3
**(MG)**	SDH (AU)	424±29	1.022±0.091	-	1.745±0.161	1.548±0.095	0.978±0.087	0.684±0.081	0.409±0.037
	GPD (AU)	424±29	0.893±0.166	-	2.424±0.119	2.837±0.187	3.232±0.291	3.549±0.189	3.919±0.183
**White**	Population (%)	647±52	0	0	0	0	7.0±1.0	4.9±0.2	88.1±1.0
**Gastrocnemius**	CSA (µm^2^)	312±22	-	-	-	-	2613.2±42.4	3806.8±66.9	5426.5±96.3
**(WG)**	SDH (AU)	312±22	-	-	-	-	0.849±0.131	0.479±0.105	0.245±0.046
	GPD (AU)	312±22	-	-	-	-	3.133±0.137	2.805±0.223	2.593±0.032
**Soleus**	Population (%)	1154±101	96.6±1.0	0.7±0.1	2.7±1.0	0	0	0	0
**(Sol)**	CSA (µm^2^)	497±28	4254.4±38.0	3553.0±59.5	3113.5±47.1	-	-	-	-
	SDH (AU)	497±28	1.000±0.058	1.524±0.127	1.780±0.128	-	-	-	-
	GPD (AU)	497±28	1.000±0.176	2.468±0.206	3.346±0.187	-	-	-	-
**Plantaris**	Population (%)	2827±211	8.3±0.9	0.5±0.1	20.5±1.5	6.4±0.3	44.6±2.6	3.2±0.2	16.5±2.0
**(Pla)**	CSA (µm^2^)	1142±37	2027.6±39.4	2387.6±47.6	2436.1±74.1	3438.3±63.3	4650.3±80.7	5992.6±131.2	6862.9±107.6
	SDH (AU)	1142±37	1.328±0.146	1.737±0.191	2.017±0.137	1.458±0.144	1.186±0.126	0.603±0.067	0.412±0.031
	GPD (AU)	1142±37	1.167±0.022	1.564±0.046	2.329±0.169	2.652±0.096	3.361±0.088	3.275±0.137	3.362±0.125
**Extensor**	Population (%)	1501±200	3.7±0.7	0.1±0.1	20.0±1.5	4.0±0.6	30.8±1.3	5.4±0.5	35.9±2.8
**Digitorum**	CSA (µm^2^)	601±44	1507.5±11.6	1848.6±20.9	1862.6±7.7	2545.7±49.3	3085.2±12.7	4073.8±13.8	5348.1±27.4
**Longus (EDL)**	SDH (AU)	601±44	1.065±0.119	1.500±0.145	1.891±0.054	1.502±0.121	1.010±0.080	0.711±0.067	0.516±0.085
	GPD (AU)	601±44	1.645±0.073	2.365±0.134	2.755±0.063	3.303±0.204	3.478±0.136	3.494±0.065	3.539±0.077
**Red Tibialis**	Population (%)	1755±85	14.3±1.4	0.5±0.1	30.3±1.5	7.4±0.4	25.2±1.5	1.7±0.2	20.7±1.4
**Anterior (RTA)**	CSA (µm^2^)	707±2	1931.3±33.5	2271.9±38.9	2155.2±34.6	2653.2±46.2	3106.9±53.2	3336.3±57.1	4233.1±71.9
	SDH (AU)	707±2	1.387±0.181	1.937±0.220	2.317±0.253	1.823±0.207	1.337±0.158	0.993±0.169	0.604±0.095
	GPD (AU)	707±2	1.071±0.078	1.657±0.211	2.410±0.210	2.894±0.170	3.178±0.146	3.547±0.185	3.745±0.179
**White Tibialis**	Population (%)	583±50	0	0	0	1.2±0.4	7.0±0.8	7.2±0.8	84.6±1.2
**Anterior (WTA)**	CSA (µm^2^)	241±4	-	-	-	3317.1±56.5	3473.8±55.9	5925.7±104.5	6527.9±116.0
	SDH (AU)	241±4	-	-	-	1.219±0.028	0.758±0.136	0.373±0.073	0.207±0.053
	GPD (AU)	241±4	-	-	-	2.283±0.070	3.165±0.139	3.240±0.060	2.961±0.206
**Vastus**	Population (%)	815±99	52.3±3.2	2.1±0.7	41.3±2.2	1.2±0.2	2.8±0.8	0.3±0.2	0
**Intermedius (VI)**	CSA (µm^2^)	417±13	4096.7±81.1	3764.9±64.4	3670.0±62.0	4231.5±83.5	4871.4±94.4	5218.2±19.2	-
	SDH (AU)	417±13	0.994±0.173	1.360±0.149	1.585±0.150	1.196±0.191	0.917±0.079	0.442±0.004	-
	GPD (AU)	417±13	1.234±0.053	1.670±0.178	2.728±0.145	3.036±0.129	3.448±0.124	3.736±0.111	-
**White Vastus**	Population (%)	648±58	0	0	0	0	2.1±0.2	2.0±0.3	95.9±0.5
**Lateralis (WVL)**	CSA (µm^2^)	278±14	-	-	-	-	4115.8±70.9	5021.9±89.0	5874.1±104.8
	SDH (AU)	278±14	-	-	-	-	0.512±0.040	0.425±0.065	0.244±0.020
	GPD (AU)	278±14	-	-	-	-	2.601±0.224	2.608±0.142	2.575±0.172

SDH and GPD activity staining are expressed relative to the values obtained for rat soleus type I fibers (assigned a reference value of 1.0). The number of fibers counted/quantified for each parameter is also given. Values are means ± SEM (n = 6 per muscle).

**Table 4:** Quantitative analysis of fiber type distribution, CSA, SDH activity, and GPD activity in mouse muscles.

			Fiber Type						
Mouse Muscle		Fiber Count	Type I	Type I/IIA	Type IIA	Type IIAX	Type IIX	Type IIXB	Type IIB
**Red**	Population (%)	681±63	7.9±0.5	0.2±0.1	41.6±1.3	5.0±0.4	19.6±2.1	3.6±0.4	22.2±1.1
**Gastrocnemius**	CSA (µm^2^)	368±43	1743.4±28.2	1300.6±21.0	1346.2±22.8	1439.1±24.7	2002.2±32.2	2476.8±39.9	2846.8±47.7
**(RG)**	SDH (AU)	368±43	1.146±0.061	1.431±0.032	1.894±0.040	1.656±0.066	1.332±0.101	1.010±0.056	0.608±0.044
	GPD (AU)	368±43	1.237±0.065	1.791±0.176	2.336±0.146	3.225±0.193	3.818±0.241	4.265±0.178	4.831±0.181
**Mixed**	Population (%)	576±68	0	0	20.9±1.6	3.6±0.4	14.8±1.1	4.9±0.8	55.7±1.4
**Gastrocnemius**	CSA (µm^2^)	268±69	-	-	1088.2±32.2	1195.2±42.3	1554.4±84.9	2281.8±70.8	2640.3±85.6
**(MG)**	SDH (AU)	268±69	-	-	1.855±0.079	1.744±0.083	1.394±0.066	1.023±0.048	0.443±0.017
	GPD (AU)	268±69	-	-	2.454±0.097	3.304±0.058	4.143±0.180	4.822±0.152	5.218±0.192
**White**	Population (%)	669±101	0	0	0	0	1.6±0.6	1.5±0.4	97.0±0.8
**Gastrocnemius**	CSA (µm^2^)	277±43	-	-	-	-	1240.7±20.0	1692.9±27.3	2639.2±49.3
**(WG)**	SDH (AU)	277±43	-	-	-	-	1.030±0.064	0.684±0.058	0.324±0.033
	GPD (AU)	277±43	-	-	-	-	3.362±0.198	3.672±0.220	4.156±0.195
**Soleus**	Population (%)	622±60	30.6±2.2	0.6±0.2	49.1±1.2	4.4±0.7	11.8±1.7	0.5±0.1	3.1±1.1
**(Sol)**	CSA (µm^2^)	259±28	1560.8±25.6	1332.8±20.3	1356.4±23.6	1550.6±53.7	1686.0±32.9	1916.4±25.1	2266.6±33.5
	SDH (AU)	259±28	1.000±0.058	1.406±0.143	1.535±0.083	1.436±0.051	1.170±0.053	0.928±0.014	0.576±0.010
	GPD (AU)	259±28	1.000±0.099	1.506±0.024	1.885±0.067	3.305±0.118	3.666±0.179	4.739±0.079	5.231±0.100
**Plantaris**	Population (%)	730±48	0	0	19.4±1.4	8.3±0.8	22.4±2.3	3.0±0.6	46.9±2.4
**(Pla)**	CSA (µm^2^)	362±38	-	-	1073.2±109.9	1222.3±107.1	1775.7±135.1	2308.0±124.6	2619.7±106.4
	SDH (AU)	362±38	-	-	1.599±0.091	1.381±0.083	1.048±0.068	0.720±0.066	0.443±0.026
	GPD (AU)	362±38	-	-	1.511±0.061	2.273±0.078	2.950±0.155	3.456±0.335	3.900±0.347
**Extensor**	Population (%)	758±59	0	0	10.2±0.9	3.6±0.5	23.7±0.9	6.8±0.6	55.7±1.2
**Digitorum**	CSA (µm^2^)	307±35	-	-	517.3±11.7	528.2±5.6	953.9±53.2	1270.9±52.1	2108.4±52.1
**Longus (EDL)**	SDH (AU)	307±35	-	-	1.495±0.124	1.384±0.089	1.027±0.054	0.775±0.056	0.356±0.022
	GPD (AU)	307±35	-	-	2.164±0.154	2.889±0.195	3.421±0.229	3.694±0.195	4.055±0.295
**Red Tibialis**	Population (%)	857±50	0.6±0.6	0	18.2±2.4	8.3±0.5	44.7±1.9	3.0±0.5	25.1±1.6
**Anterior (RTA)**	CSA (µm^2^)	368±51	1501.4±7.1	-	1369.6±22.4	1665.0±26.8	2186.0±35.2	2610.6±53.1	3073.8±51.3
	SDH (AU)	368±51	0.948±0.002	-	1.773±0.006	1.587±0.057	1.211±0.041	0.685±0.015	0.444±0.026
	GPD (AU)	368±51	1.821±0.007	-	3.122±0.133	4.015±0.144	4.694±0.165	5.161±0.209	5.568±0.202
**White Tibialis**	Population (%)	600±59	0	0	0.6±0.3	4.4±0.5	16.3±1.7	7.7±1.2	70.9±1.8
**Anterior (WTA)**	CSA (µm^2^)	249±17	-	-	735.7±31.2	998.4±46.3	1415.8±23.4	2166.4±36.2	3010.1±51.6
	SDH (AU)	249±17	-	-	1.094±0.144	0.949±0.083	0.574±0.123	0.429±0.156	0.209±0.056
	GPD (AU)	249±17	-	-	3.394±0.192	4.189±0.155	4.362±0.054	4.897±0.107	4.995±0.094
**Vastus**	Population (%)	703±54	2.4±0.5	0.3±0.2	37.6±3.2	9.4±0.6	24.7±1.3	2.1±0.5	23.5±2.5
**Intermedius (VI)**	CSA (µm^2^)	332±37	2579.3±63.4	2042.4±19.8	2087.8±29.3	2396.9±74.9	2736.2±64.6	2873.3±61.1	3732.1±102.1
	SDH (AU)	332±37	1.059±0.042	1.180±0.026	1.613±0.052	1.395±0.055	1.188±0.013	0.703±0.033	0.415±0.038
	GPD (AU)	332±37	1.161±0.028	1.485±0.175	2.179±0.145	2.872±0.124	3.279±0.085	4.065±0.124	4.245±0.154
**White Vastus**	Population (%)	542±78	0	0	0	0	1.3±0.3	5.4±0.7	93.4±0.9
**Lateralis (WVL)**	CSA (µm^2^)	218±32	-	-	-	-	913.0±25.5	1489.2±35.0	3489.8±92.0
	SDH (AU)	218±32	-	-	-	-	1.069±0.124	0.700±0.048	0.252±0.018
	GPD (AU)	218±32	-	-	-	-	3.531±0.158	3.740±0.156	3.827±0.173

SDH and GPD activity staining are expressed relative to the values obtained for mouse soleus type I fibers (assigned a reference value of 1.0). The number of fibers counted/quantified for each parameter is also given. Values are means ± SEM (n = 6 per muscle).

### Rat and Mouse Fiber CSA, SDH Activity, and GPD Activity

In general, SDH staining in rat and mouse muscle was high in type IIA fibers, intermediate in type I and IIX fibers, and low in type IIB fibers. In contrast, GPD staining intensity in rat and mouse muscle was high in type IIB or IIX fibers, intermediate in type IIA fibers, and low in type I fibers. Further, type IIB fibers tended to be the largest, type IIX fibers intermediate in size, and type IIA and I fibers the smallest. With respect to SDH activity, GPD activity, and CSA, hybrid fibers typically fell between the corresponding pure fiber types (Figures 1 & 2; Tables 3 & 4).

**Figure 4:**
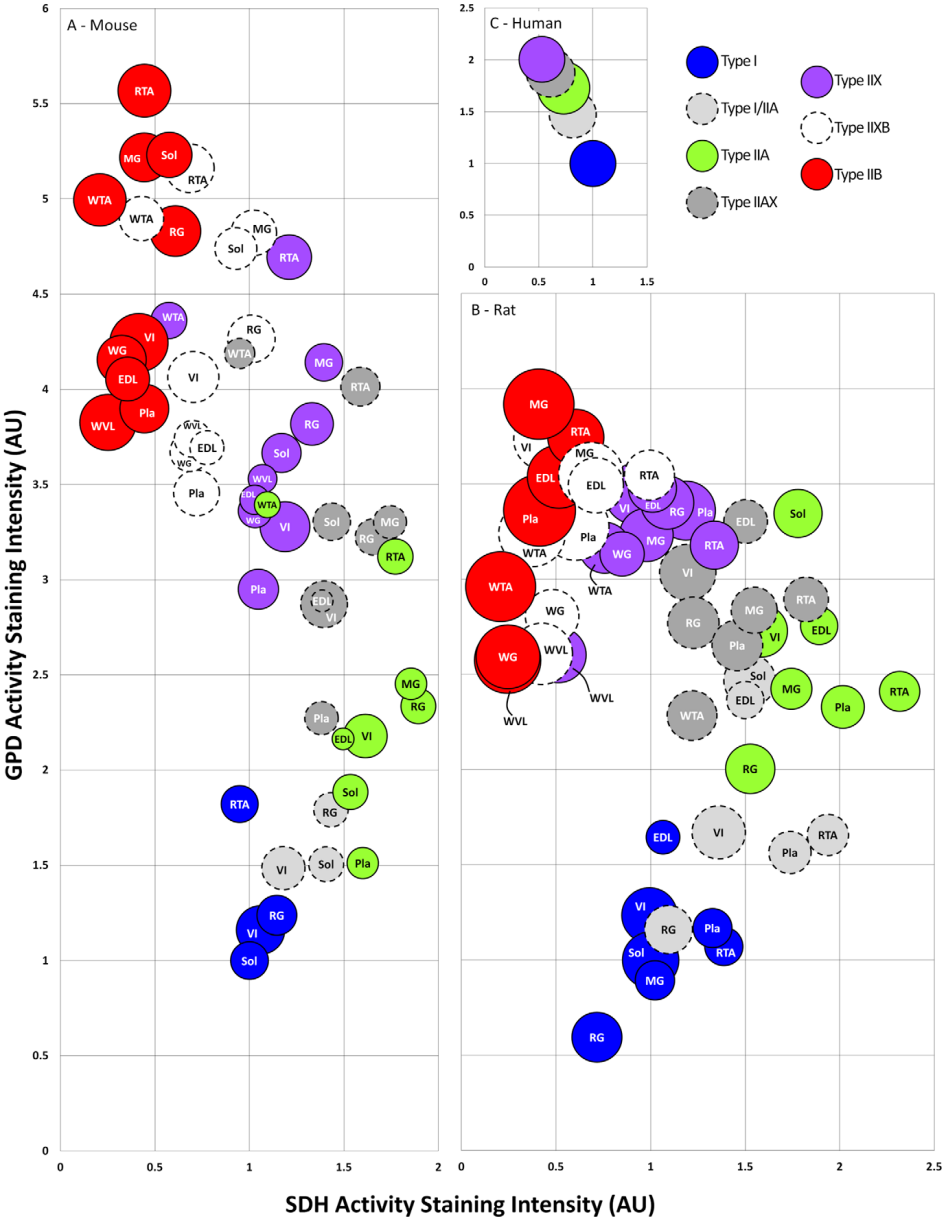
Bubble plot showing SDH activity, GPD activity, and CSA for each fiber type in mouse, rat, and human skeletal muscles. Panel A, relative fiber type-specific SDH activity, GPD activity, and CSA for ten mouse skeletal muscles. Panel B, relative fiber type-specific SDH activity, GPD activity, and CSA for ten rat skeletal muscles. Panel C, relative fiber type-specific SDH activity, GPD activity, and CSA for human VL muscle. Data presented in this figure are from Tables 3, 4, and 5. Bubble size represents the relative CSA within a species. SDH and GPD activity are expressed relative to the values obtained in type I fibers (soleus for mouse and rat, VL for human) and assigned a reference value of 1.0.

Although general fiber type trends were observed for CSA, SDH activity, and GPD activity within a particular muscle, differences in these properties were apparent for a particular fiber type across muscles (Figure 4). For example, CSA was ∼2.1-fold higher in type I fibers of rat soleus compared to rat plantaris muscle. Similarly, relative SDH activity was ∼2.7-fold higher in type IIXB fibers of rat RTA compared to rat WTA muscle, whereas relative GPD activity was ∼2.1-fold higher in type IIA fibers of mouse RTA compared to mouse plantaris muscle.

### Human Muscle Fiber Type, CSA, SDH Activity, and GPD Activity

**Figure 5:**
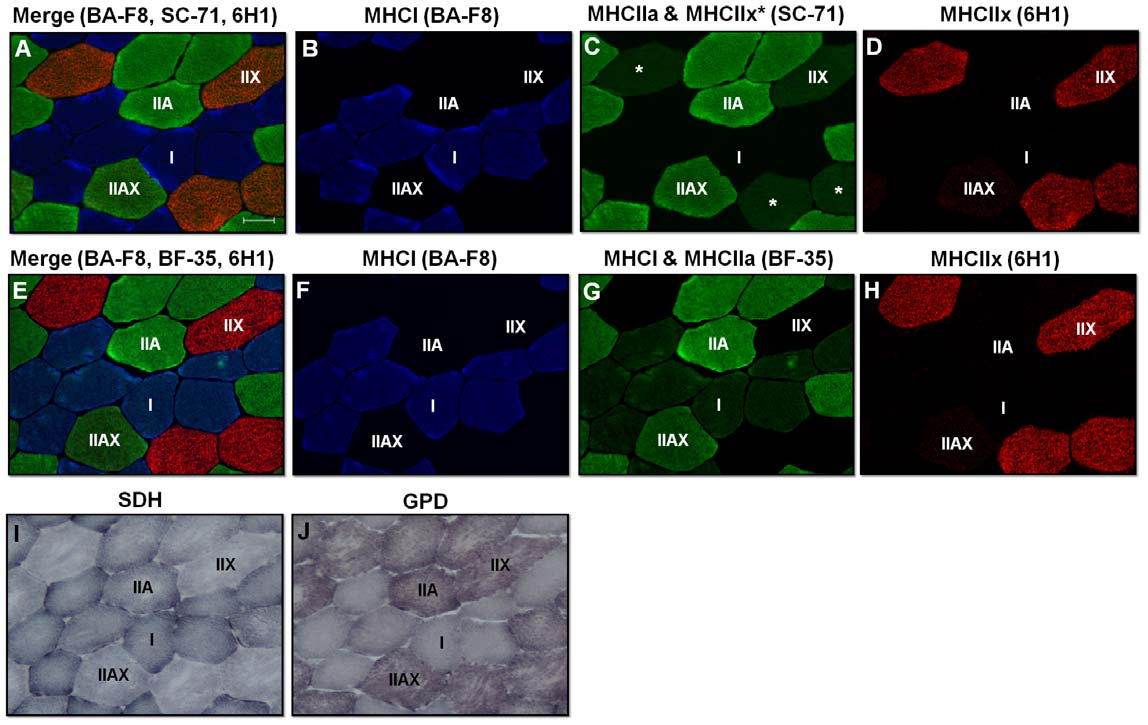
Representative images of human muscle showing MHC expression as well as SDH and GPD activity staining. Panels A–D, merged and single channel images of a human vastus lateralis (VL) muscle serial cross-section incubated with an antibody cocktail (BA-F8, SC-71, and 6H1). Shown are type I (blue), type IIA (strong green), type IIX (strong red and intermediate green), and type IIAX (intermediate/strong green and intermediate red) fibers. *Note that the lower intensity staining for SC-71 in type IIX fibers is indicative of non-specific cross-reactivity (see results and discussion for further details). Panels E–H, merged and single channel images of a human VL muscle serial cross-section incubated with an antibody cocktail (BA-F8, BF-35, and 6H1). Shown are type I (blue), type I and type IIA (intermediate green and strong green, respectively), type IIX (strong red), and type IIAX (intermediate/strong green and intermediate red) fibers. Panel I, human VL muscle serial cross-section showing SDH activity staining. Panel J, human VL muscle serial cross-section showing GPD activity staining. Bar represents 50 µm.

Incubation of human VL muscle cross-sections with various antibody cocktail combinations resulted in positive staining of the same fibers as serial cross-sections incubated with individual antibodies (*Supplementary Figure S1*). In addition, strong fluorescent staining was observed in muscle incubated with primary and secondary antibody cocktails, whereas negligible staining was observed in control slides incubated with a secondary antibody cocktail only (*Supplementary Figure S2*). Initial studies were performed with a cocktail containing BA-F8, SC-71, and 6H1. A large subset of fibers demonstrated strong staining only for BA-F8 and were classified as type I fibers. Similarly, a considerable number of fibers stained strongly only for SC-71 and were classified as type IIA fibers. A very small subset of fibers stained intermediate for both BA-F8 and SC-71 and were classified as type I/IIA fibers (Figure 3). SC-71 also intermediately stained a subset of fibers which all stained strongly for 6H1; which we initially classified as type IIX fibers. A small set of fibers was also identified that stained intermediate/strong for SC-71 and intermediate for 6H1; which we initially classified as type IIAX fibers (Figure 5).

Given that all fibers staining positive for 6H1 also stained positive for SC-71, this indicated possible non-specific cross-reactivity of the SC-71 antibody in human muscle; an effect not observed in rat or mouse muscle. Interestingly, cross-reactivity of the SC-71 antibody with human MHCIIx has been noted in several reports [21], [22]. Subsequent experiments using different pre-treatments and dilutions of SC-71 did not reduce this non-specific cross-reactivity (*data not shown*). Experiments using several dilutions of an additional antibody against MHCIIa (2F7) developed by Lucas and colleagues [15] showed similar cross-reactivity with fibers staining positive for 6H1 (*data not shown*). As such, we utilized the BF-35 antibody noted to be specific for all MHC isoforms except MHCIIx in an attempt to clearly define these fiber populations in human muscle. BF-35 stained fibers that previously stained strongly for BA-F8 and SC-71 only, corresponding to type I and type IIA fibers, respectively (Figure 5). BF-35 did not label fibers which stained strongly for 6H1 (type IIX fibers), but did label a small subset of fibers that stained intermediate for 6H1 (type IIAX fibers) (Figure 5). Using this later method, we were not able to identify type I/IIA hybrids; however, these fibers only represented 0.1% of the total population. Therefore, our previous classification of type I, I/IIA, IIA, IIAX, and IIX fibers in human VL muscle using a single incubation containing BA-F8, SC-71, and 6H1 was accurate (Figure 5 & Table 5). We quantified the fluorescence intensity of previously categorized pure and hybrid fibers and found that hybrid fibers had lower fluorescence intensity in both color channels relative to their respective pure fiber counterparts (*Supplementary Figure S3*). The fluorescence intensity of hybrid fibers was above background values, indicating that these fibers were categorized accurately. In addition, we performed optical sectioning to rule-out potential issues with out-of-focus fluorescence. Although optical sectioning by structured-illumination microscopy improved resolution compared to conventional widefield fluorescence microscopy, the ability to discriminate fiber types was similar (*Supplementary Figure S4*).

**Table 5:** Quantitative analysis of fiber type distribution, CSA, SDH activity, and GPD activity in human vastus lateralis muscle.

			Fiber Type				
Human Muscle		Fiber Count	Type I	Type I/IIA	Type IIA	Type IIAX	Type IIX
Vastus Lateralis	Population (%)	540±91	48.7±0.9	0.1±0.1	42.2±1.6	2.8±0.5	6.2±1.6
(VL)	CSA (µm^2^)	224±22	5127.8±174.0	5341.9±292.2	6616.1±209.1	5697.0±193.0	5123.3±138.1
	SDH (AU)	224±22	1.000±0.044	0.813±0.095	0.729±0.069	0.610±0.068	0.529±0.077
	GPD (AU)	224±22	1.000±0.062	1.474±0.127	1.731±0.049	1.775±0.082	2.006±0.054

SDH and GPD activity staining are expressed relative to the values obtained for human VL type I fibers (assigned a reference value of 1.0). The number of fibers counted/quantified for each parameter is also given. Values are means ± SEM (n = 7).

The relative staining intensity for SDH activity was highest in type I fibers, intermediate in type IIA fibers, and lowest in type IIX fibers. In contrast, relative staining intensity for GPD activity was lowest in type I fibers, intermediate in type IIA fibers, and highest in type IIX fibers. Fiber CSA in human muscle was largest in type IIA fibers, and smallest in type I and IIX fibers (Figures 4 & 5; Table 5).

## Discussion

A number of histological methods have been used to identify muscle fiber types [1], [8], [10], [12], [14], [16], [23], [24]. In the present investigation we outline a simple multicolor immunofluorescence procedure to identify pure and hybrid fiber types in multiple species. Compared to other methods, this procedure requires a significantly reduced time-investment due to less tissue processing (cutting of cryosections, staining) and analysis (imaging, matching, counting). Further, all major pure and hybrid fiber types could be easily identified.

A number of studies employing immunohistochemical procedures have identified type IIX fibers based on their non-reactivity with the BF-35 antibody (which stains all fibers except type IIX), due to the unavailability of an acceptable antibody against MHCIIx [14], [16], [25], [26]. We utilized the 6H1 antibody which has been shown to react with the electrophoretically separated band corresponding to MHCIIx, and labels type IIX fibers in cross-sections of a variety of species [15]. We demonstrate that the 6H1 antibody positively stained fibers that remained unstained by other antibodies in rat, mouse, and human muscle, confirming their identity as type IIX fibers. Further, this antibody correctly labelled hybrid IIAX and IIXB fibers, which displayed a lower staining intensity. A number of other reports have utilized the 6H1 antibody to identify type IIX fibers in rodent [27]–[29] and human [22] muscle. We found that a significant number of the total fibers analysed in rat (∼32%) and mouse (∼28%) muscles expressed MHCIIx, emphasizing the importance of proper identification of these fibers in rodent muscle.

Although we were limited to a three-color detection method, with the appropriate hardware (a 4-color or more microscopy system) and a modification to the present protocol, simultaneous positive-staining for all four MHC isoforms is possible on a single cross-section. In particular, antibody conjugation kits are available that allow for labelling of each primary antibody with a distinct fluorophore, thereby eliminating the need for a secondary antibody step. Further, given the increasing spectrum of available fluorophores and microscopy imaging systems, it is possible to not only identify all four MHC isoforms, but other proteins/markers of interest simultaneously on a single cross-section. It is important to note that others have previously performed multicolor analysis of MHC expression in rodent muscles [30]–[33]; however, we clearly outline a simple approach that can be used for the positive identification of all major pure and hybrid fiber populations in rat, mouse, and human muscle. Given the ease and information obtained by multicolor immunofluorescence analysis, we feel that a procedure like the one presented in this paper can be adopted as a standard protocol for identifying MHC expression in rat, mouse, and human muscle cross-sections. Interestingly, human muscle samples incubated with SC-71 (specific for MHCIIa in mice and rats) showed intermediate staining in all fibers that stained positive for MHCIIx (6H1), which is in agreement with others [21], [22]. In an attempt to clarify this issue we used an additional antibody (2F7) also noted to be specific for MHCIIa in several species [15]; however, we observed similar cross-reactivity. Smerdu and Soukup [22] found that another commercially available antibody (A7.74) noted to react with MHCIIa also cross-reacted with MHCIIx in human muscle. Further studies are needed to clarify the differential cross-reactivity of these MHCIIa antibodies in rodent and human muscle.

Although a comprehensive comparison of fiber type composition to existing literature is beyond the scope of this manuscript, the fiber type distributions of specific muscles reported here are in general agreement with previous work in rats [9], [34]–[38] and mice [25], [28], [31], [39]–[41]; however, there are notable differences across studies. Our results in humans confirm our previous report using myosin ATPase activity [17] and are in agreement with previous work [42]–[44]. We found that hybrid fibers were present in all rat and mouse muscles analysed and made up a significant proportion of most muscles. For example, rat EDL, RTA, and plantaris contained 9.5%, 9.6%, and 10.1% hybrid fibers, respectively (Table 3). Similarly, mouse EDL, RTA, and plantaris contained 10.4%, 11.3%, and 11.3% hybrid fibers, respectively (Table 4). Delp and Duan previously reported that hybrid fibers were found in some rat muscles but only made up a minor portion (<5%) of the total number of fibers. In contrast, Lucas et al. [15] found 4% hybrid fibers in the superficial region of the TA and 11% hybrid fibers in the deep region of the TA of rats. Approximately 12% of the fibers in the TA [16] and medial gastrocnemius [45] muscles of rats were reported to be hybrids. Similarly, it was found that the hybrid population ranged from 8.8% to 17.8% across five rat muscles [46]. In mice, hybrid fibers made up 4.9% of the TA muscle [16] and ranged from 18.2% to 28.7% across four muscles [47]. Collectively, these studies, along with our current report, demonstrate that hybrids account for a significant proportion of the fiber population in some rat and mouse muscles, and must be considered and properly identified in future research.

Some general relationships, which were consistent with previous reports, were observed between fiber types with respect to rat and mouse CSA [9], [25], [26], [35], [46], [48], [49], SDH activity [16], [26], [35], [45], [48]–[52], and GPD activity [26], [35], [45], [49], [51] staining. Similarly, our human CSA, SDH activity, and GPD activity data are in general agreement with previously published literature [44], [53]–[55]. Collectively, this data provides further confirmation that the MHC immunofluorescence procedure outlined in this report is effective in properly identifying pure and hybrid fibers in multiple species. Although general relationships have been noted for morphological and metabolic properties within fibers of a particular MHC composition, variability exists. For example, it has been previously demonstrated that SDH activity, GPD activity, and CSA were related to MHC composition; however, large variation existed between individual fibers within rat medial gastrocnemius muscle [45], [49]. It is possible that some of the variation in these properties within a muscle is due to differences in the muscle portion isolated and/or sampling position; however, we followed the scheme outlined by Armstrong and Phelps [11], and consistently cryo-sectioned at the mid-portion of muscles. In addition, any variability due to sampling position was minimized in the present investigation by quantifying a large portion (over 40%) of the fibers within a muscle/cross-section. We were also able to examine the general size and metabolic characteristics of a particular fiber type across a number of rat and mouse muscles (Figure 4). In general, although fibers of a particular MHC composition showed similar characteristics, notable differences were observed in CSA, SDH activity, and GPD activity between different muscles. Several factors likely contribute to these findings including anatomical and recruitment differences between muscles. Regardless, this data reinforces the notion that fibers of the same MHC composition can show considerable differences in morphological and metabolic properties within and between muscles.

In conclusion, we describe a simple and efficient method for identifying pure and hybrid fibers in rat, mouse, and human muscle cross-sections using commercially available MHC-specific antibodies and immunofluorescence analysis. We suggest that the procedure presented herein will be useful for determining MHC expression and performing fiber type level analysis in rodent and human muscle. In addition, we provide a useful resource of the pure and hybrid fiber type distribution (along with fiber CSA and relative SDH and GPD activity) for a number of rat and mouse muscles.

## Supporting Information

Figure S1 **Representative images of skeletal muscle cross-sections showing positive staining of the same fibers following incubation with individual antibodies versus antibody cocktails.** Panels A–E (rat RTA), F–J (mouse RG), K–O (human VL). Corresponding images were captured using identical exposure parameters within each channel. Bars represent 50 µm.
(TIF)

Figure S2 **Representative images of skeletal muscle cross-sections showing typical positive staining compared to background staining.** Panels A, C, and E are cross-sections from rat, mouse, and human muscles, respectively, incubated with only fluorescent-conjugated secondary antibody cocktails. Panels B, D, and F are serial cross-sections incubated with primary antibody cocktails (BA-F8, SC-71, and BF-F3 for rat and mouse; BA-F8, SC-71, and 6H1 for human) followed by incubation with fluorescent-conjugated secondary antibodies. Corresponding images were captured using identical exposure parameters. Bars represent 50 µm.
(TIF)

Figure S3 **Quantification of fluorescence staining intensity of previously categorized pure and hybrid fibers.** Fluorescence quantification in rat muscle (Panel A), mouse muscle (Panel B), and human muscle (Panel C). Note that due to the cross-reactivity of the SC-71 antibody with type IIX fibers in human muscle, the green fluorescence in type IIX fibers is not negligible. However, the green fluorescence due to this cross-reactivity in the type IIX fibers is lower than the green fluorescence obtained in both the pure type IIA and hybrid type IIAX fibers. Fluorescence in pure fibers is assigned an arbitrary value of 1.0, with hybrid fibers expressed relative to corresponding pure fibers. Values shown are means ± SEM (n = 2 per species).
(TIF)

Figure S4 **Comparison of fluorescent images acquired by widefield versus structured illumination microscopy.** Representative images of a human muscle cross-section incubated with an antibody mixture (BA-F8, SC-71, and 6H1, followed by secondary antibodies) captured using conventional widefield (Panels A–D), or optical sectioning via structured illumination (Panels E–H) fluorescence microscopy. *Note that the lower intensity staining for SC-71 in the fiber also staining positive for 6H1 (MHCIIx) is indicative of non-specific cross-reactivity (see results and discussion for further details). Bars represent 50 µm.
(TIF)
