# Supplementary figures and images for: Rapid Determination of Myosin Heavy Chain Expression in Rat, Mouse, and Human Skeletal Muscle Using Multicolor Immunofluorescence Analysis
Source: PLoS One. 2012 Apr 18;7(4):e35273. doi: 10.1371/journal.pone.0035273 (PMC3329435; doi:10.1371/journal.pone.0035273)

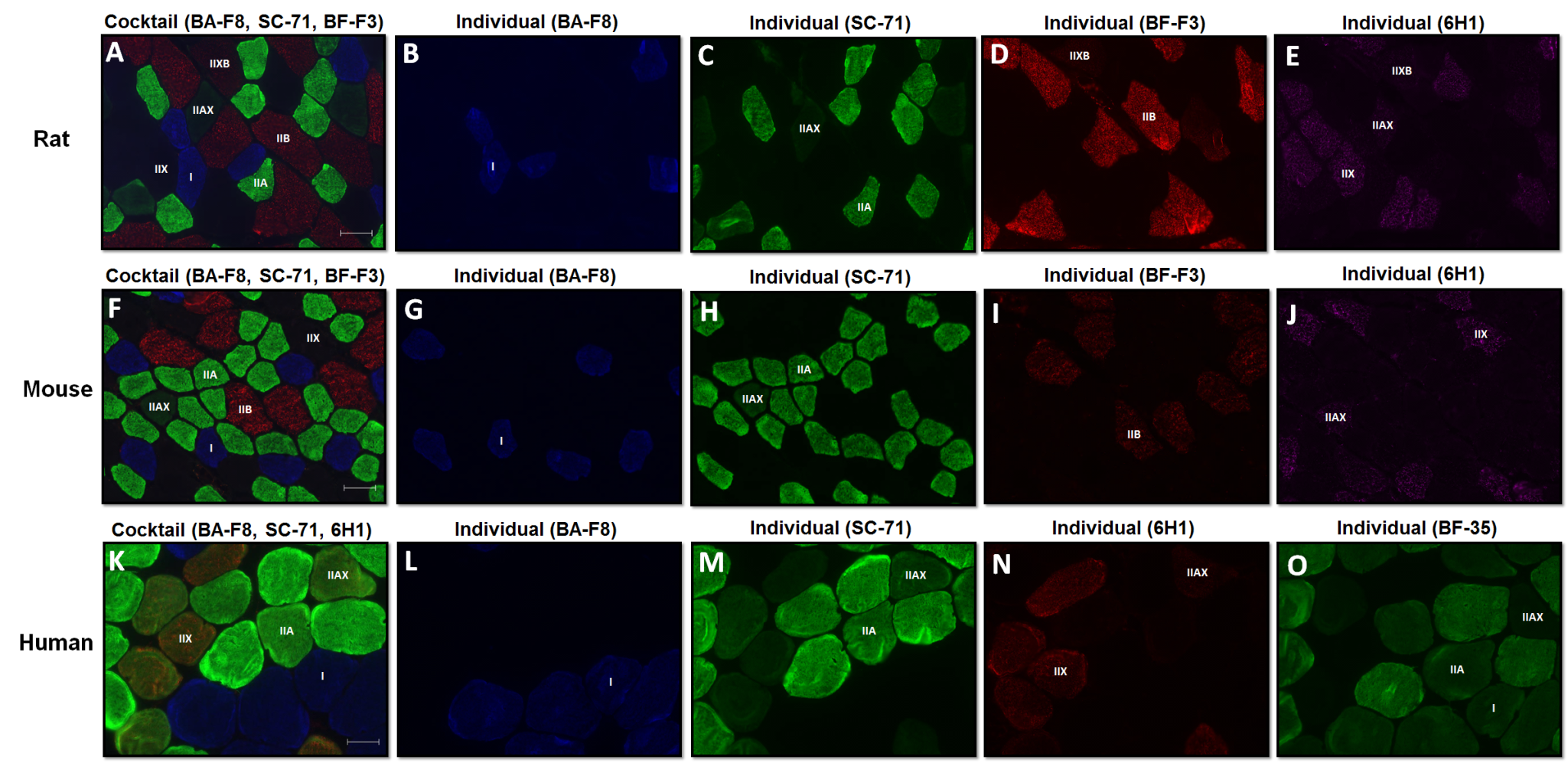

Supplement: Figure S1 — Representative images of skeletal muscle cross-sections showing positive staining of the same fibers following incubation with individual antibodies versus antibody cocktails. Panels A–E (rat RTA), F–J (mouse RG), K–O (human VL). Corresponding images were captured using identical exposure parameters within each channel. Bars represent 50 µm. (TIF) [file pone.0035273.s001.tif]

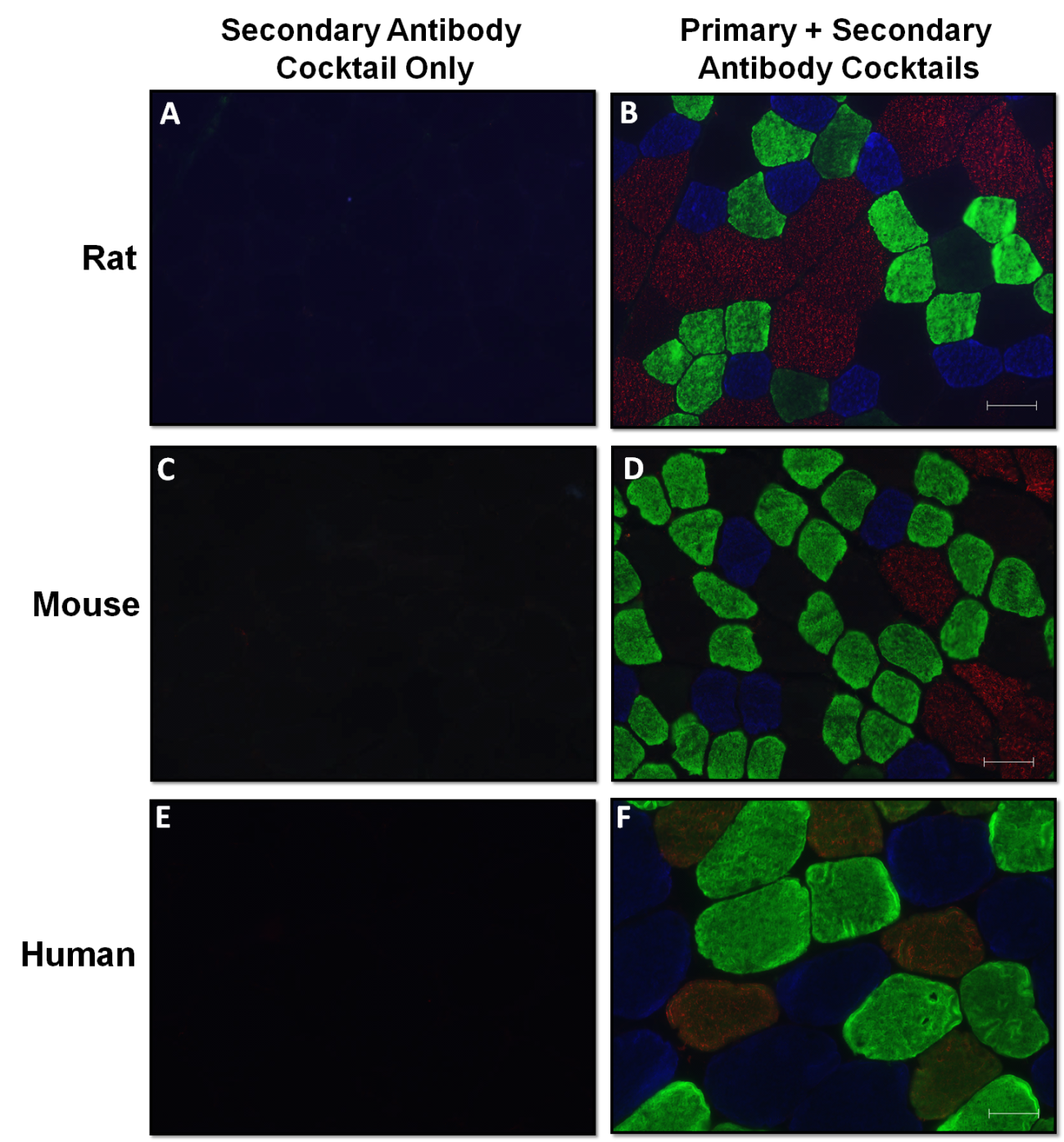

Supplement: Figure S2 — Representative images of skeletal muscle cross-sections showing typical positive staining compared to background staining. Panels A, C, and E are cross-sections from rat, mouse, and human muscles, respectively, incubated with only fluorescent-conjugated secondary antibody cocktails. Panels B, D, and F are serial cross-sections incubated with primary antibody cocktails (BA-F8, SC-71, and BF-F3 for rat and mouse; BA-F8, SC-71, and 6H1 for human) followed by incubation with fluorescent-conjugated secondary antibodies. Corresponding images were captured using identical exposure parameters. Bars represent 50 µm. (TIF) [file pone.0035273.s002.tif]

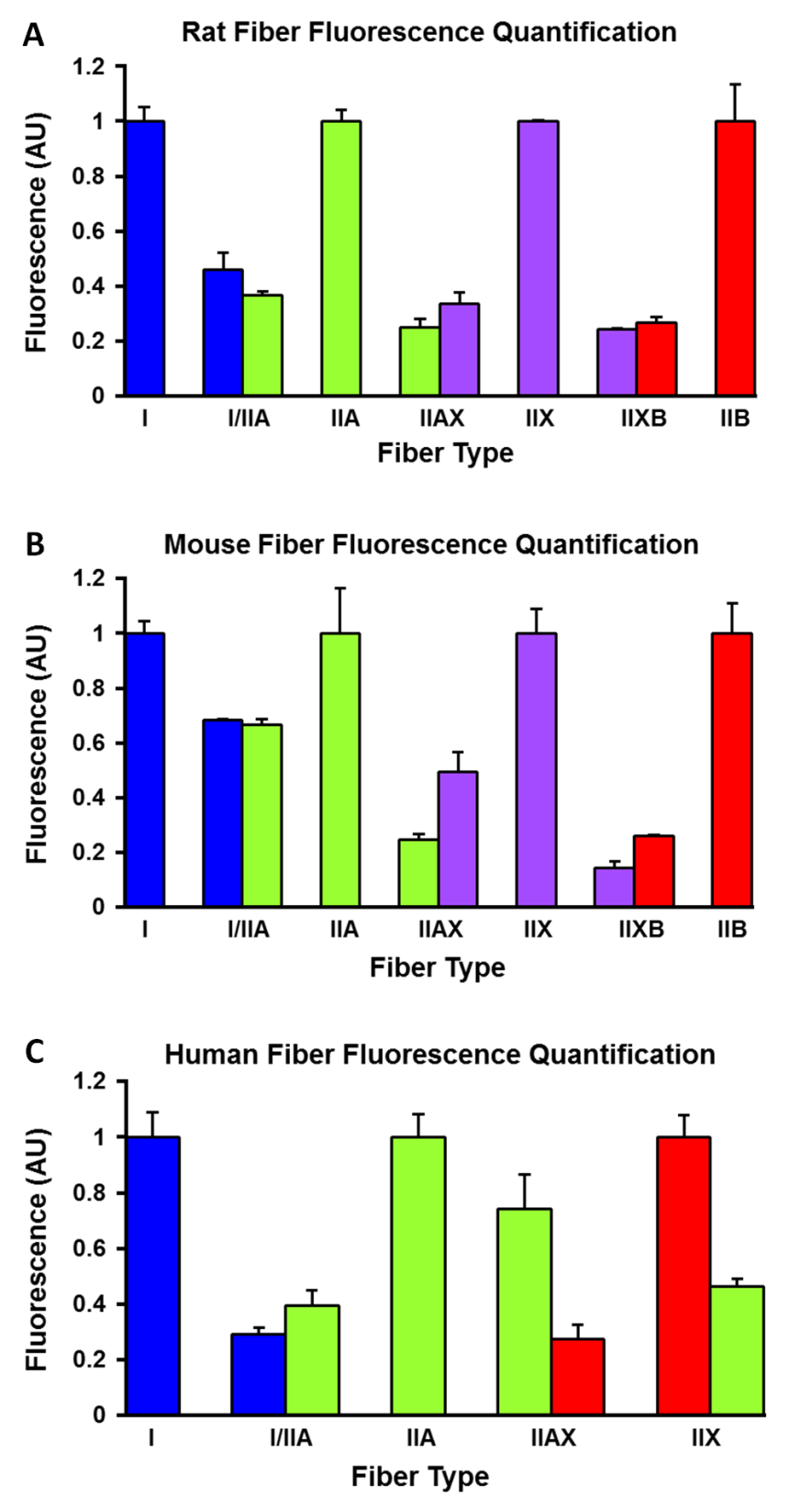

Supplement: Figure S3 — Quantification of fluorescence staining intensity of previously categorized pure and hybrid fibers. Fluorescence quantification in rat muscle (Panel A), mouse muscle (Panel B), and human muscle (Panel C). Note that due to the cross-reactivity of the SC-71 antibody with type IIX fibers in human muscle, the green fluorescence in type IIX fibers is not negligible. However, the green fluorescence due to this cross-reactivity in the type IIX fibers is lower than the green fluorescence obtained in both the pure type IIA and hybrid type IIAX fibers. Fluorescence in pure fibers is assigned an arbitrary value of 1.0, with hybrid fibers expressed relative to corresponding pure fibers. Values shown are means ± SEM (n = 2 per species). (TIF) [file pone.0035273.s003.tif]

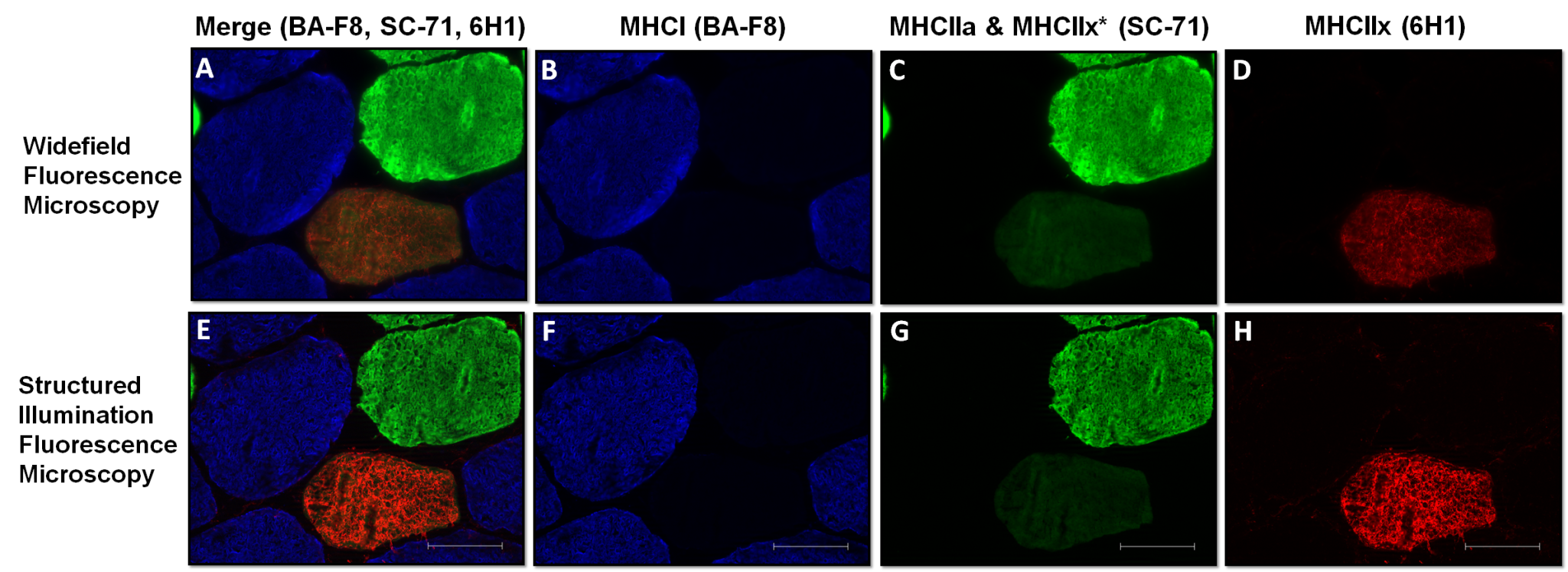

Supplement: Figure S4 — Comparison of fluorescent images acquired by widefield versus structured illumination microscopy. Representative images of a human muscle cross-section incubated with an antibody mixture (BA-F8, SC-71, and 6H1, followed by secondary antibodies) captured using conventional widefield (Panels A–D), or optical sectioning via structured illumination (Panels E–H) fluorescence microscopy. *Note that the lower intensity staining for SC-71 in the fiber also staining positive for 6H1 (MHCIIx) is indicative of non-specific cross-reactivity (see results and discussion for further details). Bars represent 50 µm. (TIF) [file pone.0035273.s004.tif]
